# Surgical Outcomes of Hybrid-Robotic Compared with Non-Robotic Oncological Esophagectomy for Adenocarcinoma Using a Fail-Safe Protocol—A Cohort Study

**DOI:** 10.3390/cancers18111820

**Published:** 2026-06-01

**Authors:** Jonas Herzberg, Matilda Bariani, Tim Strate, Salman Yousuf Guraya, Human Honarpisheh

**Affiliations:** 1 Department of Surgery, Krankenhaus Reinbek St. Adolf-Stift, 21465 Reinbek, Germany; 2 College of Medicine, Gulf Medical University, Ajman P.O. Box 4184, United Arab Emirates

**Keywords:** Ivor Lewis esophagectomy, anastomotic leakage, robotic surgery esophageal cancer

## Abstract

**Background**: Ivor Lewis esophagectomy for esophageal malignancies is a core element of the treatment but still comes with substantial postoperative complications. Especially within the learning curve of robotic esophagectomy, an optimal treatment pathway is crucial to minimize the clinical impact of this phase. This study aimed to compare the surgical outcomes of hybrid-robotic esophagectomy for adenocarcinoma of the esophagus with non-robotic procedures using a fail-safe protocol. **Methods**: This retrospective single-center study evaluated the outcome of hybrid-robotic procedures within the early robotic learning curve in comparison to a historical control group of laparoscopic and open non-robotic procedures using uni- and multivariable regression analysis. CUSUM analysis was applied for learning curve outcomes. All procedures were performed between January 2016 and December 2025 within a standardized fail-safe approach. The primary end point was the occurrence of anastomotic leakage (AL), whereas the overall complications as well as the number of harvested lymph nodes were secondary end points. **Results**: A total of 156 patients were analyzed, including 50 hybrid-robotic and 106 non-robotic resections. Robotic surgery was associated with a higher lymph node yield (40.9 ± 11.0 vs. 35.7 ± 13.1; *p* = 0.011) and a lower lymph node ratio (0.085 vs. 0.125; *p* = 0.046). The AL rate was 4.0% in the robotic group and 5.7% in the non-robotic group. Length of stay was significantly shorter after robotic procedures (13.1 ± 5.5 vs. 18.6 ± 11.3 days; *p* < 0.001). **Conclusions**: Establishing a robotic esophagectomy program within an established structured fail-safe protocol was not associated with an increased risk of anastomotic leakage or major complications. Within this standardized setting, an increased lymph node yield, potentially optimizing oncological quality was observed. These findings indicate that acceptable preoperative safety and oncological outcomes can be achieved even during the learning curve of hybrid-robotic esophagectomy embedded in a structured fail-safe protocol.

## Simple Summary

Esophageal surgery still comes with a high risk for postoperative complications. Robotic-assisted esophagectomy can improve postoperative recovery and outcome in cancer patients. The learning curve of robotic procedures still comes with a rise in postoperative complications. This study evaluated the outcome of 50 hybrid-robotic esophagectomies within a multimodal fail-safe concept during the learning curve of robotic esophageal surgery. These data were compared with 106 standard laparoscopic and open oncological esophagectomies using the same perioperative fail-safe approach. The robotic group showed a shorter length of stay and a higher number of removed lymph nodes. The anastomotic leakage rate showed no significant difference between the two groups (4.0% vs. 5.7% in the non-robotic group). These findings suggest that introducing a robot-assisted esophageal surgery program within the framework of an existing, structured fail-safe protocol does not increase the risk of serious complications even during the learning curve. The higher lymph node yield in the hybrid-robotic group may additionally indicate a potentially improved oncological quality of surgery.

## 1. Introduction

In esophageal cancer treatment, esophagectomy remains the cornerstone [1]. Ivor Lewis esophagectomy with transthoracic reconstruction using a gastric tube forms the established approach for tumors in the distal esophagus and in the gastroesophageal junction. As this is a challenging operative technique that involves an abdominal and a thoracic approach, the postoperative complication rate remains high [2]. One of the perilous complications is the anastomotic leakage (AL) of the esophago-gastric anastomosis. International literature continues to report an AL incidence of up to 30% [3,4]. This severe complication has the potential to exert a deleterious effect on the postoperative course, as evidenced by prolonged postoperative stay necessitated by the administration of additional treatments such as endoscopic procedures or reoperation. Moreover, it has been demonstrated that this complication can affect the long-term outcome of the patients and has the potential to increase the risk of postoperative short-term mortality [5,6].

This high incidence of complications led to numerous enhancements in the perioperative care considering several technical aspects. The existing data had shown a correlation between caseload and postoperative morbidity [7,8], so to improve the perioperative environment, a centralization was implemented. A series of additional perioperative measures was made to minimize the risk of postoperative complications. Among others, the optimization of perioperative nutrition was considered, as this showed an impact on postoperative outcomes, particularly the AL rate [9,10], as well as the prophylactic use of CPAP (continuous positive airway pressure) to reduce postoperative pneumonia as previously reported [11].

In our institution all these enhancements were integrated into a multimodal fail-safe concept, reducing not only the rate of severe postoperative events but also their impact on the postoperative course. The concept was initially established in the context of colorectal cancer surgery [12] and pancreatic cancer surgery [13] and has been adopted for esophageal cancer surgery, resulting in an AL rate of 12.6% as previously published [14].

In addition to these developments, the surgical technique has been and continues to be a primary focus to further improve postoperative outcomes. For example, this led to the implementation of additional tools and instruments like the use of intraoperative fluorescence angiography to guarantee a sufficient blood supply within the anastomotic region [15]. The most drastic change within the last few years was the shift in surgical approach from open toward a less invasive method, like a minimally invasive technique, and now to a robotic-assisted surgery, showing an improved patient outcome [16,17,18,19]. As robotic surgery evolves to represent a paradigm shift in surgical methodology, even experienced esophageal surgeons must undergo a period of adjustment to master this new approach [20,21]. Current literature reports a learning curve of around 50 cases to reduce postoperative complications [20].

The aim of this study was to compare the surgical outcomes of hybrid-robotic and non-robotic esophagectomy for adenocarcinoma of the esophagus using a fail-safe protocol during the learning curve.

## 2. Materials and Methods

This is a retrospective cohort study comparing hybrid-robotic Ivor Lewis esophagectomies with a historical non-robotic cohort from the study center, including laparoscopic and open non-robotic Ivor Lewis resections. The medical records of all patients who underwent oncological surgical resection of the esophagus between January 2016 and December 2025 at Krankenhaus Reinbek St. Adolf-Stift, Germany, were evaluated. All patients with an Ivor Lewis resection for adenocarcinoma of the esophagus or the esophagogastric junction were included. Patients with other surgical procedures than Ivor Lewis resections and those with Ivor Lewis procedures for other indications, such as squamous cell cancer or benign lesions, were excluded.

The preoperative work-up was performed in accordance with the German guidelines for esophageal cancer treatment [19]. This included endoscopy, endosonography, and a computed tomography (CT) scan of the thorax and abdomen. All patients were thereafter reviewed by a multidisciplinary tumor board (MDT), referring the patient to neoadjuvant treatment or to upfront surgery in accordance with the national guidelines [22]. After identification of patients meeting the inclusion criteria, their medical records were reviewed for medical history, preoperative parameters (BMI, ASA), multimodal treatment, and surgical parameters (technique, duration of surgery, and pathological examination, including tumor staging using the Union for the International Cancer Control (UICC) classification). In addition, all patients with at least one positive lymph node were included, calculating the lymph node ratio (LNR) as the number of positive lymph nodes within the resected lymph nodes [23]. Postoperative outcomes were analyzed for the length of hospital stay and occurrence of postoperative complications as classified by Dindo–Clavien’s classification [24]. The anastomotic leakage (AL) was defined as radiologically and/or endoscopically confirmed leakage from the anastomosis [25]. In case of an AL, the treatment regimen was reviewed. Postoperative pulmonary complications such as pneumonia were defined by a typical clinical presentation combined with systemic inflammation (elevated leucocytes or fever) and a corresponding radiological finding [26].

### 2.1. Surgical Work-Up

**Figure 1:**
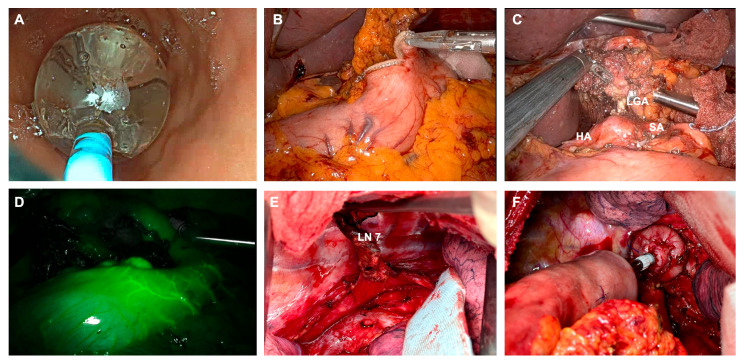
Critical surgical steps during the hybrid robotic procedures within the fail-safe approach. (**A**): Preoperative dilatation of the pylorus using a balloon. (**B**): First cut forming the gastric tube. (**C**): After mobilization showing the coeliac trunk with the hepatic artery (HA), the splenic artery (SA) and the left gastric artery (LGA) before cutting the LGA. (**D**): Fluorescence angiography representing a good blood supply by the gastroomental artery. (**E**): After right thoracotomy and removal of lymph nodes, Level 7 (LN7). (**F**): Performing the end-to-side circular stapling anastomosis.

In our study, all patients were treated by a standardized fail-safe approach as reported for colorectal and pancreatic cancer surgery in the same institution [12,13]. As a part of this fail-safe protocol, all patients prior to esophageal resection received a nutritional work-up and parenteral nutrition the day before surgery. Our nutrition regimen included 1000 mL of crystalloids the day before surgery. One day prior to surgery, an endoscopic pyloric dilatation up to 2 cm was carried out by a balloon (Figure 1). A preoperative single-shot antibiotic treatment using 1500 mg cefuroxime and 500 mg metronidazole was administered right before the incision. At our center, the resection was performed as a hybrid approach wherever possible [9,27]. This included a laparoscopic gastric mobilization preserving the gastroepiploic vessels. After implementing a robotic surgery program for esophageal cancer surgery in 2023 in the study center, the abdominal phase was performed robotically using the daVinci Xi system (Intuitive Surgical Operations, Inc., Sunnyvale, CA, USA). After the abdominal part, including an additional three-field lymphadenectomy, the thoracic part was performed by right-sided thoracotomy in all groups. The term ‘hybrid-robotic’ refers specifically to a robotic abdominal phase combined with an open thoracic phase. The thoracic phase consisted of an en bloc transthoracic esophagectomy along with the mediastinal lymph node dissection. In advance of anastomosis, an intraoperative fluorescence angiography was done using ICG (Figure 1). The intrathoracic anastomosis was performed either as a side-to-side linear stapling or as an end-to-side circular stapling technique, depending on the availability of different stapling devices during the study period. Additional sutures were performed to secure the stapled anastomosis. After formation of the anastomosis, a nasogastric tube was inserted, and an air leakage test was conducted.

**Figure 2:**
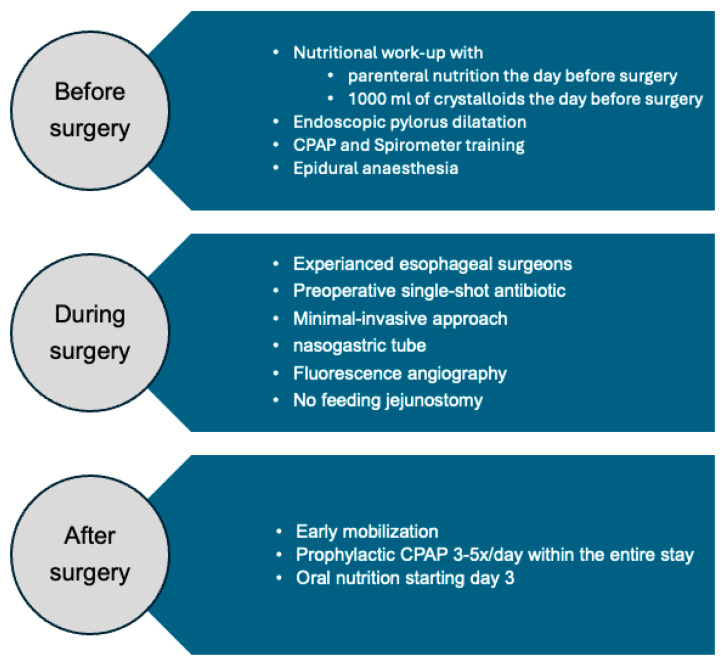
Perioperative fail-safe work-up.

All patients were treated at least 24 h after surgery in an intensive care unit. All patients received parenteral nutrition, beginning with oral intake on the third postoperative day with clear fluids. No feeding tube or other enteral access was used in this protocol (Figure 2).

All procedures were performed by two experienced surgeons, who had completed their learning curve in esophageal surgery before this study period. All robotic procedures were performed by one of these experienced esophageal surgeons.

### 2.2. Statistical Analysis

IBM SPSS Statistics Version 31 (IBM Co., Armonk, NY, USA) was used for the statistical analysis. The Kolmogorov–Smirnov test was used to test for normal distribution. Linear variables were listed as means with standard deviation; categorical variables were arranged as numbers with percentages. Relationships between categorical variables were tested using the chi-square test. If the linear variables were normally distributed, a *t*-test was used. In case of non-normal distribution, the non-parametric Mann–Whitney U test was done. In addition, a regression analysis was conducted to evaluate the impact of robotic surgery on the postoperative outcome. A 95 confidence interval is reported, and a *p*-value < 0.05 was considered statistically significant.

In the multivariate analysis, we used logistic regression to predict the occurrence of postoperative complications in robotic surgery. The sample size for logistic regression was determined using the rule of at least 10 events per variable as reported by Peduzzi et al. [28]. Multicollinearity was assessed using variance inflation factors (VIFs). A *p*-value less than or equal to 0.05 was considered statistically significant. A linear regression analysis was conducted to assess the impact of robotic surgery on postoperative length of stay.

To investigate the learning curve, a cumulative sum (CUSUM) analysis of the operative time, length of stay and postoperative major complications was performed using IBM SPSS Statistics Version 31 (IBM Co., Armonk, NY, USA). In this graphical method, raw data were transformed into a running total of differences from the group average as described in several studies before [29,30]. For major complications, the benchmark reported by Low et al. of 31% was used [25].

This study was conducted in accordance with the Declaration of Helsinki and approved by the Ethics Committee of the Medical Association of Schleswig-Holstein. The database forming the source for this retrospective study was prospectively registered in the German Clinical Trial Registry (Study-ID: DRKS00029753). Data are reported in accordance with the STROBE guidelines [31].

## 3. Results

**Figure 3:**
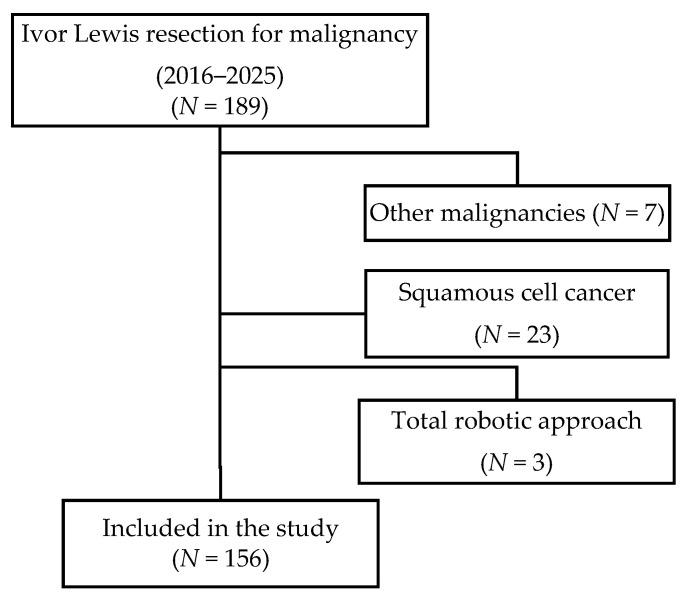
A flow chart of all patients with different types of esophageal malignancies in our study.

Between January 2016 and December 2025, 189 patients were treated by Ivor Lewis resection for esophageal malignancy in our study center. This included 156 patients with adenocarcinoma of the esophagus (Figure 3).

Based on the Shapiro–Wilk test, the length of hospital stay, duration of surgery, and BMI showed no normal distribution. There were no missing data in key variables.

We found that 139 (89.1%) of the included patients were male, with a mean age of 66.2 ± 10.0 years. While 50 patients received a hybrid robotic procedure, 106 patients were treated without robotic surgery. This included 61 minimally invasive procedures (laparoscopic approach combined with thoracotomy) and 45 patients treated with a complete open technique. The patients’ characteristics are listed in Table 1.

The number of female patients in the robotic group was higher than in the non-robotic group. Apart from that, the patients’ and tumor characteristics did not differ between the two groups. The number of patients treated with neoadjuvant radio-chemotherapy was higher in the non-robotic surgery group (13.2% vs. 0.0%; *p* = 0.007).

**Figure 4:**
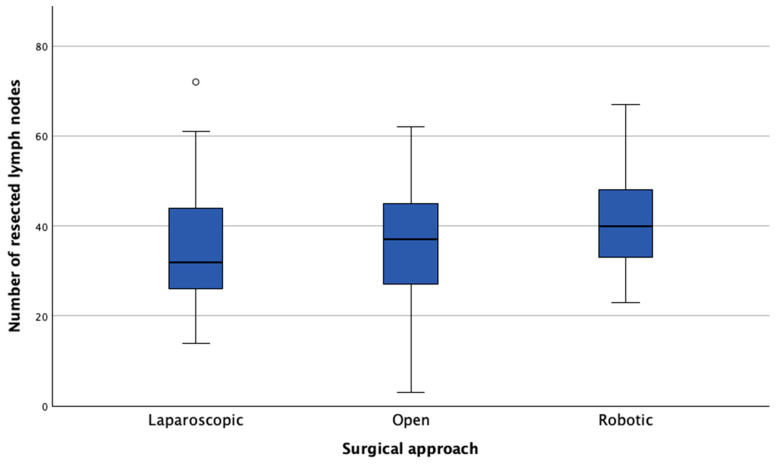
Box-and-whisker plot showing the number of resected lymph nodes depending on the surgical approach; the robotic approach yielded significantly more lymph nodes (*p* = 0.011). Boxes indicate the interquartile range, central lines the median, whiskers the non-outlier range, and the dot represents an outlier (the outlier showed 72 resected lymph nodes within the laparoscopic approach).

The hybrid-robotic approach increased the number of resected lymph nodes significantly in comparison to the non-robotic group (40.9 ± 11.0 vs. 35.7 ± 13.1; *p* = 0.011). (Figure 4).

The number of positive lymph nodes did not differ significantly between the two groups (*p* = 0.960). In 73 patients, no lymph node involvement was detected with a scored lymph node ratio (LNR) of 0. The LNR in the pN+ patients was significantly lower within the robotic group compared with non-robotic procedures (0.085 vs. 0.125; *p* = 0.046; 95% CI 0.0007–0.0805). In the robotic group, the rate of postoperative complications was significantly less compared to the non-robotic group (Dindo-Clavien >1. Robotic: 44% vs. 57.5 non-robotic; *p* < 0.001). However, there was no significant difference for major complications between the two groups (Dindo-Clavien > 3a). In the robotic collective, we detected an AL of 4% during the learning curve, whereas this was 5.7% in the non-robotic group. This difference did not reach the level of significance. In addition, the rate of postoperative pneumonia was reduced in the hybrid-robotic group but did not reach the level of statistical significance (*p* = 0.213). In a subgroup analysis comparing minimally invasive procedures with the hybrid-robotic approach, the results remain consistent with a reduced pneumonia rate in robotic procedures (28% vs. 39.3%) without statistical significance (*p* = 0.233).

**Figure 5:**
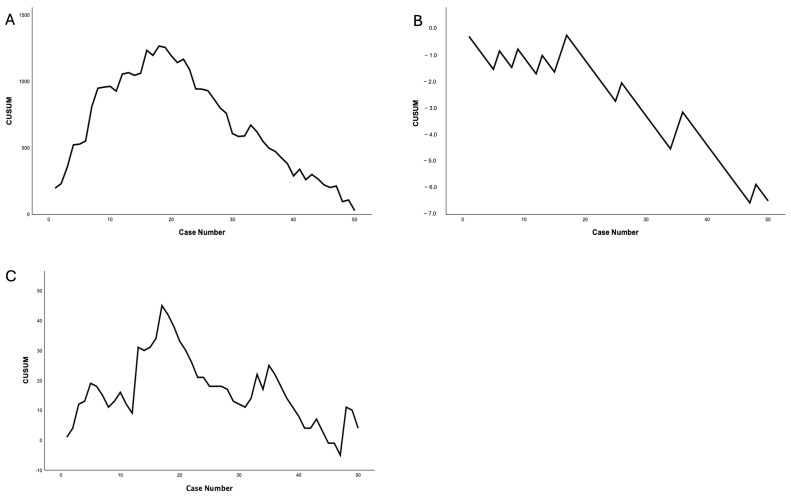
CUSUM analysis for the hybrid-robotic learning curve. (**A**): Duration of surgery. (**B**): Postoperative major complication Dindo–Clavien > 3a. (**C**): Postoperative length of stay.

An additional learning curve analysis was performed using the CUSUM method. The duration of surgery increased during the initial phase, underlining a longer operation time at the beginning of the learning curve (Figure 5A). A turning point was observed after case 19, followed by a downward slope indicating increasing procedural efficiency and technical stabilization.

Postoperative major complications (Dindo–Clavien > 3a) remained below the international reported benchmark of 31% throughout the learning curve. The CUSUM curve showed a further decline from case 20 onwards (Figure 5B). Similarly, the CUSUM curve for postoperative length of stay descended from case 18 (Figure 5C).

Following logistic regression, including surgical approach as a three-level categorical variable (open, laparoscopic, robotic), robotic surgery was associated with lower odds of postoperative complications compared with conventional laparoscopic surgery, although this did not reach statistical significance. For the control variables, preoperative ASA score was the only factor associated with the occurrence of at least one postoperative complication (OR 3.182, 95% CI 1.611–6.284, *p* < 0.001) (Table 2). All VIF values were low (1.05–1.61), indicating no relevant multicollinearity among the predictors.

For a sensitivity analysis, patients with a neoadjuvant CROSS treatment were excluded, as these were only present in the control group. In this analysis of 142 patients, robotic surgery was associated with significantly lower odds of postoperative complications compared with laparoscopic surgery (OR 0.38, 95% CI 0.152–0.963, *p* = 0.041).

These beneficial effects of robotic surgery resulted in a significantly shorter length of stay (13.1 ± 5.5 days vs. 18.6 ± 11.3 days; *p* < 0.001), even within the subgroup analysis excluding open procedures (13.1 ± 5.5 days vs. 18.2 ± 11.8 days in laparoscopic surgery; *p* = 0.006).

This positive effect of robotic surgery was also consistent within a linear regression analysis. After adjusting for age, sex, ASA, and tumor stage, patients after robotic surgery had a 4.787-day shorter stay (95% CI –7.933 to –1.641; *p* = 0.003) (Table 3). Also in this analysis, all VIF values were low (1.03–1.1), indicating no relevant multicollinearity. The occurrence of a postoperative complication led to a prolonged postoperative stay of 8.61 days (*p* < 0.001) according to the multivariate regression analysis.

The postoperative 30-day mortality was higher within the hybrid-robotic cohort (6.0% vs. 0.9%), even so this difference did not reach the level of significance. The three patients who died following a hybrid robot-assisted procedure were hybrid-robotic case number 6, who died of postoperative cardiac arrest, and cases 35 and 36, who died of complications resulting from AL; treatment was discontinued early on for one of these patients at the request of the family.

## 4. Discussion

Our study demonstrates early results of hybrid-robotic oncological esophagectomy within a structured multimodal fail-safe protocol with favorable perioperative outcomes even during the early learning curve. The hybrid-robotic approach was associated with a significantly higher lymph node yield and a shorter postoperative hospital stay without increasing the risk of AL or major postoperative complications. The observed AL rate of 4% during the hybrid-robotic learning phase is notably lower than rates commonly reported in contemporary literature. An additional strength of our study is the incorporation of CUSUM learning-curve analysis, which validated a progressive procedural stabilization and improved efficiency after approximately 20 surgical cases. These findings reaffirm the feasibility of safe integration of advanced surgical technologies within an established multidisciplinary perioperative approach.

In our study, both hybrid-robotic and standard groups were comparable regarding baseline characteristics. Only the rate of women was higher in the robotic group.

In the robotic group, no patient received neoadjuvant radio-chemotherapy. This is in line with the current literature. As reported within the ESOPEC trial in 2025, the neoadjuvant chemotherapy following the FLOT protocol comes with an improved overall and disease-free survival in comparison to neoadjuvant radio-chemotherapy [32]. This change towards a neoadjuvant chemotherapy, which has been favored in the study center even before the publication of the ESOPEC trial, can explain the low rate of neoadjuvant radio-chemotherapy in the robotic group. The logistic regression showed a trend towards a reduced rate of postoperative complications after neoadjuvant FLOT treatment, although this was not statistically significant. This is in line with data presented by Bhimani et al., reporting a significantly lower rate of postoperative complications of 38% after neoadjuvant chemotherapy in comparison to upfront surgery (57%) or neoadjuvant radio-chemotherapy (57%; *p* = 0.009) [33]. The fact that no patient in the robotic group received neoadjuvant radio-chemotherapy might lead to residual confounding affecting postoperative morbidity and AL rates despite multivariable adjustment. To address this, a sensitivity analysis was performed, excluding patients after CROSS treatment in which the association between robotic surgery and reduced postoperative complications remained statistically significant (*p* = 0.041). As this analysis only includes 142 of the 156 patients, the statistical power is reduced and it should be interpreted as a robustness analysis. Further studies treating patients with esophageal adenocarcinoma following the ESOPEC results are needed.

In this analysis, the number of resected lymph nodes was significantly increased within the robotic group (40.9 ± 11.0 vs. 35.7 ± 13.1; *p* = 0.011). A lymph node harvest of more than 15 lymph nodes is a quality metric in esophageal cancer surgery, as a lymph node dissection of fewer than 15 lymph nodes is associated with a poor survival rate [34]. Several studies underlined the benefit of robotic surgery for lymphadenectomy [35]. A large meta-analysis evaluating the effect of robotic esophageal surgery showed a significant increase in harvested lymph nodes in the robotic group, whereas the reported mean numbers of 28.89 and 26.61, respectively, are below the rate reported here [36]. Apart from this, a recently published analysis showed that not only the number of harvested lymph nodes but also the lymph node ratio is important to achieve a good oncological result with an optimal overall survival [37]. In this analysis, the lymph node ratio after robotic procedures is significantly lower than after non-robotic procedures (0.085 vs. 0.125; *p* = 0.046; 95% CI 0.0007–0.0805). In accordance with the grouping presented by Liang et al. in 2023, the non-robotic procedures also showed a lymph node ratio below 0.23 [38]. A lower lymph node ratio is identified to correlate with a better overall and disease-free survival [23,37].

The rate of AL was 4% in the hybrid-robotic group and 5.7% in the non-robotic group. Although this difference did not reach statistical significance, both rates are below the data published in the current literature, reporting AL rates between 11.43% [36] and 24.4% [3]. The very low AL rate in our cohort, especially in the robotic group, might reflect a small sample size, and therefore it should not be generalized by principle, especially as a large series from the US showed robotic esophagectomy to be associated with an increased AL rate [39]. The low AL rate in both groups might reflect the effectiveness of the reported fail-safe approach as we demonstrated previously regarding colorectal and pancreatic surgery [12,13].

Jeon et al. reported a change point for improvement of AL after 50 procedures in their series of 500 esophagectomies [20]. Our data shows that even within the first 50 cases following a structured perioperative management protocol, a low rate can be achieved. As these data represent an early learning curve, a change in the outcome may occur with gaining experience.

Overall, the rate of major complications did not differ significantly between the robotic and the non-robotic cohorts, being in line with the current literature [40]. According to the CUSUM analysis and, in comparison with the benchmark data reported by Low et al., the rate of major complications was below the benchmark of 31% even within the early phase of the learning curve [25]. Current data from another German center reported a major complication rate of 41.7% in the early phase, which is far above the rate achieved using the fail-safe protocol [29]. In the logistic regression, ASA was the only factor associated with an increased risk for postoperative complications (*p* = 0.001). This is in line with the literature presenting the ASA status as an independent predictor for postoperative complications [41].

Postoperative pneumonia showed a trend towards a lower rate in robotic surgery, but this did not reach the level of significance. The meta-analysis from Perry et al. did not identify a significant difference in pulmonary complications in robotic esophageal surgery [36]. Hauge et al. presented in their retrospective analysis an increased risk for postoperative pneumonia (OR 2.3; *p* < 0.01) in robotic esophagectomy [42]. In our cohort, the thoracic part was performed by thoracotomy and therefore did not differ between the groups. The trend towards a lower rate within the robotic group might be influenced by the inclusion of open procedures in the control group. These conventional procedures in the upper abdomen are known to come with a high risk for postoperative pneumonia [43].

In this study, the postoperative length of stay was significantly shorter in the robotic group (13.1 ± 5.5 days vs. 18.6 ± 11.3 days; *p* < 0.001). This was confirmed within the linear regression analysis, demonstrating a 4.8-day shorter length of stay after robotic surgery (*p* = 0.003). According to the CUSUM analysis, the turning point for the length of stay was 18 cases, which is comparable to current literature [29]. The inflection points in CUSUM analyses may be influenced by changes in patient selection during the learning curve and may not simply reflect the progression of the surgical training.

The beneficial effect of robotic esophageal surgery was demonstrated in several studies [36,40,44]. The duration of surgery was prolonged in the hybrid-robotic procedures, whereas this difference was not statistically significant. The CUSUM analysis showed a decrease in operation time from case 19 onwards, which is also in line with current literature [29]. A recently published meta-analysis with more than 18,000 patients showed a median length of stay of 19 days in the robotic group and 33 days in the minimally invasive group (*p* < 0.0001) [36]. This is much longer than in the cohort presented in our study, especially as our data include conventional esophagectomy, underlining the effect of the multimodal fail-safe concept even in non-robotic surgery.

The observed difference in 30-day mortality between the robotic and non-robotic groups should be interpreted with caution, given the low event numbers and the inherent limitations of retrospective analyses. Although no statistically significant difference was detected, the higher proportion of early postoperative deaths in the robotic cohort highlights the importance of careful patient selection during the early adoption phase of a new surgical technique. Several meta-analyses showed no impact of the surgical approach on the postoperative mortality in general [36,45]. The reported rate in the study and control group is in line with data from a large meta-analysis of high-volume centers of 5–6% [46]. Nevertheless, a potential influence of the learning curve and case selection during the initial implementation of robotic surgery cannot be excluded. Therefore, these findings should be interpreted in the context of a developing program.

### Limitation

This study is limited by its retrospective single-center design and the relatively small number of hybrid-robotic procedures included. This might have influenced the low rate of postoperative AL and the increased mortality rate in the robotic group. As the learning curve in robotic Ivor Lewis resection is known to be around 80 procedures, this reflects only a part of the learning curve. For AL and postoperative mortality, acknowledging the low number of events is important, as this limits the statistical power and entails a considerable risk of type II error. The robotic results might be improved as they are compared to a historical cohort. The absence of statistically significant differences should not be overinterpreted and needs approval in larger cohorts. The control group displays not only minimally invasive procedures but also open esophagectomies which could have biased the results despite the performed subgroup analysis and adjustments. Therefore, the results from this study should be confirmed in a prospective randomized trial.

## 5. Conclusions

This study reports the outcome of esophagectomy for esophageal adenocarcinoma within a multidisciplinary fail-safe approach. Within this standardized framework, the learning curve of robotic surgery was not associated with increased anastomotic leakage and was accompanied by a shorter hospital stay. Additionally, a higher lymph node yield was reported in the hybrid-robotic group without increased complications, suggesting a potential oncological benefit. However, as all patients were treated within the same structured protocol, no causal conclusions can be drawn regarding the specific role of the fail-safe protocol affecting the learning curve in the hybrid-robotic esophagectomy group. Nevertheless, the CUSUM analyses showed comparable or superior data in an international comparison. Cost, training, and operative time remain deterrents to a wider adoption of robotic surgery.

## Figures and Tables

**Table 1:** Study group characteristics values in numbers (%) except where otherwise specified. (*N* = 156). Bold letters refer to a *p* < 0.05. Non-robotic group includes hybrid-laparoscopic and open procedures, whereas robotic group includes hybrid-robotic procedures. In all groups, the thoracic phase was done by thoracotomy.

Features	Total *N* = 156	Non-Robotic Group *N* = 106	Robotic Group *N* = 50	*p*-Value
Age, M ± SD [years]	66.2 ± 10.0	66.3 ± 10.0	66.1 ± 9.8	0.914 ^a^
Sex				0.004 ^b^
Male	139 (89.1)	100 (94.3)	39 (78.0)	
Female	17 (10.9)	6 (5.7)	11 (22.0)	
ASA classification				0.205 ^c^
ASA 1	6 (3.8)	3 (2.8)	3 (6.0)	
ASA 2	68 (43.6)	42 (39.6)	26 (52.0)	
ASA 3	79 (50.6)	58 (54.7)	21 (42.0)	
ASA 4	3 (1.9)	3 (2.8)	0 (0.0)	
BMI, M ± SD [kg/m^2^]	26.3 ± 6.8	26.1 ± 5.4	26.7 ± 8.6	0.944 ^d^
Preoperative treatment				
FLOT	111 (71.2)	74 (69.8)	37 (74.0)	0.590 ^c^
CROSS	14 (9.0)	14 (13.2)	0 (0.0)	**0.007 ^c^**
None	30 (9.2)	17 (16.0)	13 (26.0)	0.191 ^b^
Other	1 (0.6)	1 (0.9)	0 (0.0)	1.000 ^c^
pTNM stage				
pT*				0.195 ^c^
pT0	13 (8.3)	9 (8.5)	4 (8.0)	
pT1	28 (17.9)	14 (13.2)	14 (28.0)	
pT2	22 (14.1)	16 (15.1)	6 (12.0)	
pT3	85 (54.5)	60 (56.6)	25 (50.0)	
pT4	8 (5.1)	7 (6.6)	1 (2.0)	
pN*				0.737 ^c^
pN0	73 (46.8)	50 (47.2)	23 (46.0)	
pN1	33 (21.2)	23 (21.7)	10 (20.0)	
pN2	40 (25.6)	25 (23.6)	15 (30.0)	
pN3	10 (6.4)	8 (7.5)	2 (4.0)	
R0 resection	148 (94.9)	101 (95.3)	47 (94.0)	0.712 ^c^
Duration of surgery [min]	402.2 ± 83.0	393.6 ± 80.8	420.5 ± 85.3	0.319 ^d^
Number of resected lymph nodes, M ± SD	37.4 ± 12.7	35.7 ± 13.1	40.9 ± 11.0	**0.011 ^a^**
Number of positive lymph nodes, M ± SD	2.1 ± 3.3	2.3 ± 3.7	1.7 ± 2.1	0.960 ^d^

M: Mean. SD: Standard Deviation. ASA: American Society of Anesthesiologists. BMI: Body mass index. ^a^ *t*-test. ^b^ Fisher’s exact test. ^c^ Chi-square test. ^d^ Mann–Whitney U test.

**Table 2:** Binary logistic regression evaluating the impact on the occurrence of postoperative complications (*N* = 156). χ^2^(7, *N* = 156) = 26.351, *p* < 0.001. Nagelkerke R^2^: 0.209. Hosmer–Lemeshow test, χ^2^(8) = 9.788, *p* = 0.208. Bold letters refer to a *p* < 0.05. ASA: American Society of Anesthesiologists. Lap: laparoscopic abdominal approach.

Variable	Coeff.	Std. Error	Wald	Exp(B)	95% CI	*p*-Value
Age	0.019	0.020	0.897	1.019	0.980–1.060	0.344
Sex	0.928	0.625	2.208	2.530	0.744–8.604	0.137
ASA	1.158	0.347	11.112	3.182	1.611–6.284	**<** **0.001**
Approach			2.640			0.267
Open vs. Lap	−0.390	0.487	0.640	0.677	0.261–1.759	0.424
Robotic vs. Lap	−0.717	0.445	2.588	0.488	0.204–1.169	0.108
T-stadium	−0.080	0.172	0.218	0.923	0.659–1.292	0.641
FLOT	−0.885	0.533	2.758	0.413	0.145–1.173	0.097
CROSS	−0.957	0.784	1.489	0.384	0.083–1.787	0.222
Constant	−3.569	1.709	4.359	0.028		**0.037**

**Table 3:** Linear regression evaluating the impact of robotic surgery on the postoperative length of stay (*N* = 156). Adjusted R^2^: 0.242; F (5, 150) = 10.896, *p* < 0.001. Bold letter refers to a *p* < 0.05.

Variable	B	Std. Error	β (Beta)	t	95% CI	*p*-Value
Robotic	−4.787	1.592	−0.220	−3.007	−7.933–−1.641	**0.003**
T	−0.525	0.664	−0.056	−0.791	−1.837–0.786	0.430
Age	0.075	0.073	0.073	1.026	−0.070–0.221	0.306
Sex	0.417	2.357	0.013	0.177	−4.240–5.074	0.860
Postoperative Complication	8.610	1.487	0.420	5.792	5.673–11.547	**<0.001**
Constant	9.176	5.804	-	5.792	−2.293–20.644	0.116

## Data Availability

The data presented in this study are available on request from the corresponding author.
